# Control of a Multi-Drug-Resistant *Acinetobacter baumannii* Outbreak after Orthopedics Department Relocation

**DOI:** 10.3390/microorganisms1010158

**Published:** 2013-12-02

**Authors:** Vasiliki Gogou, Georgios Meletis, Dimosthenis Tsitouras

**Affiliations:** 1 Department of Clinical Microbiology, Veroia General Hospital, Veroia 59100, Greece; E-Mail: vasilikigogou@yahoo.com; 2 Orthopedics Department, Veroia General Hospital, Veroia 59100, Greece; E-Mail: dennistsitouras@hotmail.com

**Keywords:** *Acinetobacter baumannii*, outbreak, orthopedics

## Abstract

*Acinetobacter baumannii* clinical isolates have the ability to survive in the hospital niche for prolonged time periods and to develop resistance against multiple antimicrobial agents. Therefore, *A. baumannii* has emerged as an important cause of nosocomial outbreaks worldwide, especially in critical-care environments such as intensive care units. In the present communication, we report a multi-drug-resistant *A. baumannii* outbreak that occurred in an orthopedics department in Greece after the admission of a patient previously hospitalized in the intensive care unit of a Greek tertiary care hospital. Despite the implementation of infection control measures, 29 patients were infected, significantly raising their hospitalization periods and treatment costs. Interestingly, the outbreak was put under control after the department’s previously programmed relocation.

## 1. Introduction

Outbreaks caused by Multi-Drug-Resistant **(**MDR) *Acinetobacter baumannii* consider mainly burn and intensive care units (ICUs) around the world. Particularly in Greece, carbapenem resistance among gram negative nosocomial pathogens has become a major public health problem in the last decade [1]. Several outbreaks of carbapenem resistant *A. baumannii* infections have been described in tertiary care hospitals, mainly due to OXA (Oxacillinase-Class D β-lactamase)-carbapenemase-producing isolates [2,3,4] whereas VIM (Verona Integron-encoded Metallo-β-lactamase)-producers have also been detected sporadically [5,6,7]. We report here an *A. baumannii* outbreak in the orthopedic department of Veroia General Hospital, a regional hospital in Northern Greece, which was put under control only after the department’s relocation.

## 2. Outbreak Description and Laboratory Procedures

The first MDR *A. baumannii* isolation was made in our hospital in 2007 from a patient of the orthopedic department; however, isolation rates of the microorganism remained low until the beginning of the outbreak in 2010. The aforementioned suspected index case was a colonized patient who was admitted from the ICU of a Greek tertiary care hospital.

Despite progressive implementation of multiple infection control measures including patient isolation, personnel hand hygiene control and thorough material disinfection, a total of 29 patients were infected with MDR *A. baumannii* over a 2-year period from June 2010 until June 2012. All the patients affected were hospitalized for intertrochanteric and subtrochanteric fractures and underwent intramedullary-nailing surgery, while one of them was subjected to total hip replacement surgery. The age of the patients ranged from 70 to 90 with average an age of 83 years. Eleven of them were male and 18 were female.

The identification and susceptibility testing of the clinical isolates were performed by Vitek 2 (bioMérieux, Marcy l’Etoile, France). Phenotypic detection for the production of KPC and/or metallo-β-lactamases was applied for all carbapenem resistant isolates using the combination meropenem disc test [8] but resulted negative. Susceptibility rates were 56% and 50% for imipenem and meropenem, respectively. Moreover, the isolates showed 100% sensitivity to colistin, 74% to gentamycin, 100% to minocycline, 57% to ampicillin/sulbactam, 85% to tigecycline, 21% to moxifloxacin and 4% to cefepime. All other antibiotics tested were resistant *in vitro.*

The clinical manifestations of these cases were serious wound infections that increased hospitalization time from the routine 6, up to approximately 20 days (330%) raising similarly the hospitalization cost and the subsequent rehabilitation period. Of note, is the four times surgical debridement that underwent the patient who was subjected to a total hip replacement in order to evade a revision surgery. This pattern extended the patient’s hospitalization from 6 days to 6 months.

Surveillance cultures were obtained from the hospital environment (orthopedic department and surgery room, from non-infected patients, and from healthcare providers). The organism was recovered from the traction table and the suction device in the surgery room and from washbasins from the patient’s rooms, suggesting that environmental contamination has played an important role in the outbreak establishment, probably together with other risk-factors such as surgical interventions to elderly patients and hand transmission.

**Figure 1:**
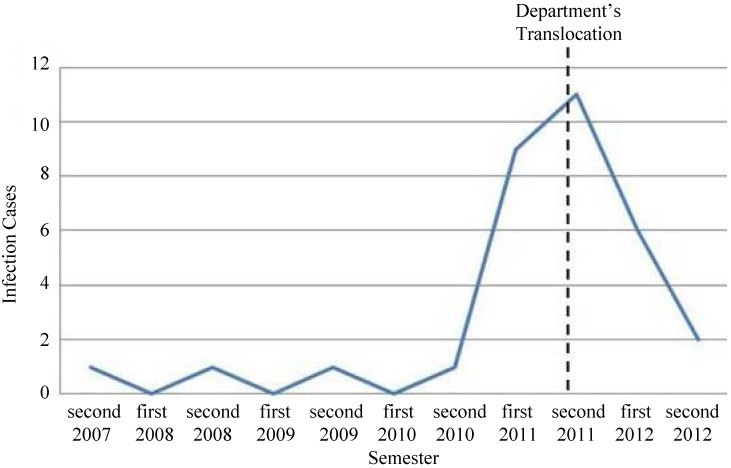
*A. baumannii* infection cases until the beginning of each year’s first and second semester.

Interestingly, the infection cases of *A. baumannii* significantly decreased after a programmed department relocation in a newly built section of the hospital in April 2011. In fact, the number of patients affected was 21 before and 8 after the relocation (Figure 1).

## 3. Conclusions

Outbreaks of *A. baumannii* usually belong to a limited number of clonal lineages, which are called International clone I, II and III corresponding to CC1, 2 and 3 respectively [9]. *A. baumannii* strains in Greece were susceptible up to the 1990s. At the beginning of 2000s a low level resistance to carbapenems led to the predominance of MDR strains in the hospitals [10]. The main carbapenemase harbored in Greek strains is OXA-58 [11] with a shift to OXA-23 after 2009 [12] which explains our negative combination meropenem disc test results. Most of the outbreaks in Greece belong to CC1 and CC2 clones, while the emergence of strains that belong to less common clones underlines the evolutionary success of *A.* *baumannii* in Greece [11]. In our case, the isolation of the microorganism in the department’s environment before, and its diminished prevalence in patient infections after the relocation shows the important role of object colonization in outbreaks due to MDR *A. baumannii*. Overall, our report highlights the propensity of *A. baumannii* to colonize the hospital environment and the serious eradication issues that concern an outbreak establishment by this pathogen.
